# A refined approach of the tachypacing porcine model of heart failure

**DOI:** 10.3389/fcvm.2026.1726438

**Published:** 2026-03-02

**Authors:** Leonhard Berboth, Jens Ötvös, Alessandro Faragli, Beatrice De Marchi, Gianluigi Longinotti-Buitoni, Paul Steendijk, Philipp Attanasio, Burkert M. Pieske, Heiner Post, Frank Heinzel, Francesco Paolo Lo Muzio, Alessio Alogna

**Affiliations:** 1 Department of Cardiology, Angiology and Intensive Care Medicine, Deutsches Herzzentrum der Charité, Berlin, Germany; 2 Berlin Institute of Health at Charité—Universitätsmedizin Berlin, Berlin, Germany; 3 DZHK (German Centre for Cardiovascular Research), partner site Berlin, Berlin, Germany; 4 L.I.F.E. Italia s.r.l., Milan, Italy; 5 Department of Cardiology, Leiden University Medical Center, Leiden, Netherlands; 6 Department of Cardiology, Campus Benjamin Franklin, Charité—Universitätsmedizin Berlin, Berlin, Germany; 7 Department of Medicine and Surgery, University Hospital of Parma, Parma, Italy

**Keywords:** 3Rs, data analysis, dilated cardiomyopathy, HFrEF, jacketed external telemetry

## Abstract

**Background:**

Preclinical models of heart failure (HF) play a key role in developing new therapeutic strategies. Tachypacing is the gold standard to induce dilated cardiomyopathy (DCM) with reduced ejection fraction (EF) in large animals, but it is not exempted from failures and can induce relevant stress.

**Aim:**

Establishing a revised porcine model of tachypacing-induced HF to improve reliability and reduce stress on the animals.

**Methods:**

Eight (*n* = 8) females Göttingen minipigs were divided in two groups: 4 animals were implanted a right ventricular two-lead pacemaker to induce HF via tachypacing, while 4 animals without implant served as controls. After a recovery period, pigs were paced asynchronously at 180 bpm for 2-weeks and 200 bpm for 4-weeks. Disease progression was assessed by echocardiography, while hemodynamics was measured invasively before sacrifice. Stress was evaluated by jacketed external telemetry (JET), cortisol, body weight, and clinical symptoms.

**Results:**

Echocardiographic assessment showed that all paced animals developed stable DCM as demonstrated by increase of end-systolic and end-diastolic volume at highly depressed ejection fraction. Invasive measurements confirmed these results with stable mAOP despite impaired pump function. JET showed no alterations of respiratory rate and daily activity throughout the protocol. Cortisol and cortisone levels and body weight showed no significant differences between groups or during pacing.

**Conclusions:**

We established a reliable model of tachypacing-induced HF based on slower pacing and milder progression to HF, while reducing the stress and suffering of the animals.

## 1 Introduction

Heart failure (HF) is a worldwide pandemic. In 2017, approximately 64,3 million people suffered from this condition (1) and its prevalence in developed countries is estimated at 1%–2% of the general adult population (2), increasing the risk of death more than 5-fold (3). After a substantial improvement in HF survival during the early 1990s, it seems this effect has levelled off thereafter (4), yet the global burden of HF is projected to increase due to ageing population and shift toward high-fat, high-sugar diets and sedentary behavior (5). This demonstrates the ongoing need to develop further treatment options. Large animal models can be very helpful to accurately characterize the effects of novel agents and therefore play an important role in the translation from bench-to-bedside.

As recently reviewed by Charles et al., there are different large animal models of HF, which can be classified in models that lead to either a phenotype of heart failure with reduced or preserved ejection fraction (HFrEF and HFpEF, respectively) (6). The two main and well-established models of HFrEF are the myocardial infarction and chronic rapid pacing. In the first, an acute myocardial ischemia is performed to induce chronic HF during the trial. This can be achieved in dogs, sheep, and pigs in an open- or closed-chest approach, for instance by coronary artery ligation or microembolization. Despite the advantage of modelling the most frequent underlying cause of HFrEF (2), this approach comes with risks of lethal arrhythmia, low reproducibility, and often leading to only a moderate left ventricle (LV) dysfunction (6).

The second approach is tachypacing (i.e., chronic rapid pacing) induced HFrEF. This model has been first described in the 1970s (7) and is based on pacing the heart at a rate 2–4-fold higher than physiological heart rate (HR). Since then, it has become the gold standard of dilated cardiomyopathy (DCM) as it induces a low-output biventricular dysfunction with dilated ventricles, reduced LV wall thickness and contractility (8). Moreover, it is accompanied by typical neurohumoral alterations present in HF (9). However, a period of compensated HF is traditionally followed by a shorter phase of decompensating HF during which animals can suffer severe symptoms including anorexia, lethargy, weight loss, dyspnea, cyanosis, and rarely sudden death (10–12).

In this work, we established a revised porcine model of tachypacing-induced HF, based on slower pacing and milder disease progression, to reduce the suffering in accordance with the principle of refinement in animal trials (13). To monitor symptoms, we developed a novel non-invasive jacketed telemetry system that can be used to monitor vital parameters in large laboratory animals.

## 2 Methods

This animal trial is approved by the Landesamt für Gesundheit und Soziales (LAGeSo) Berlin under the permission G 0064/19 and conforms to the Guide for the Care and Use of Laboratory Animals published by the U.S. National Institutes of Health (NIH publication no. 85-23, revised 1996). The data was acquired in the context of the CUPIDO Project (H2020-NMBP-2016 720834) which aimed to develop and test inhalable nanoparticles to deliver a mimetic peptide as a therapy for HFrEF. In that trial (14), animals were assigned to three groups: control, HF, and treatment. In this work, we excluded the treatment group to focus only on the revised model of tachypacing-induced HF and included only the Controls and HFrEF animals that were followed with the developed JET system (*n* = 4 per group).

After arriving at the facility, animals were vaccinated against mycoplasma and circovirus (Mycoflex®, Circoflex®) followed by a 4-week quarantine, to qualify their immune system for the trial facility. During this time, they had restricted daily contact to a small number of persons such as animal caretakers and veterinarians. At the same time, the daily training started to achieve their trust for later handling.

### 2.1 Pacemaker implantation

Four females Göttingen minipigs (Ellegaard Göttingen Minipigs, Dalmose, DK; 29 ± 2 kg; aged 10–18 months) were sedated with Midazolam (0,3 mg/kg), Xylazine (2%; 5,3 mL/30 kg), Ketamine (20 mg/kg) and atropine (1%; 1 mL). Premedication was administered into the neck muscles caudal to the ear, starting with Midazolam and Atropine directly followed by Ketamine and Xylazine. First effects of sedation were seen after 4–5 min. After 10–12 min the animal was transported to the operating room.

Proper anesthesia was initiated through an indwelling catheter placed into an auricular vein and induced by a bolus of Propofol (2%, 2–5 mg/kg) and Fentanyl (5 µg/kg) to allow for intubation with an endotracheal tube. A volatile anesthesia with Florene® (1,0–1,2 Vol%) combined with Fentanyl (35 µg/kg/h) was used. Breathing volume was set to 8 mL/kg with an FiO_2_ of 0.5 and an I:E ration of 1:1.5. The respiratory frequency was adjusted to keep the end-expiratory partial pressure of carbon dioxide (PaCO_2_) between 35 and 40 mmHg. Electrocardiogram (ECG) and O_2_-saturation were measured constantly during surgery. The animal was positioned in the supine position and covered with blankets to prevent loss of body temperature. Front limbs were tied to the operating table under light suspension to stabilize the animal and maintain access to the operating field. The incision line between the sternum and mandibular bone was widely shaved, disinfected, and covered under sterile conditions.

Ampicillin/Sulbactam (3 g i.v.) was administered to prevent postoperative infection.

The implantation of the pacemaker is comparable to the technique used for humans. A small incision (approximately 3–4 cm) was made on the right ventral side, using the sternocleidoid muscle as reference point. The right inner jugular vein was prepared and trans jugular access established. Two leads were introduced to the apical right ventricle under fluoroscopic guidance through a 9F percutaneous lead introducer hooked in the myocardium. Leads were positioned in a S-curved shape to avoid dislocation due to animal growth and correct positioning was ensured by impedance measures with sufficient distance between both electrodes. Then, the pacemaker was placed subcutaneously near the sternocleidoid muscle by forming a pocket, followed by surgical closing of the wound.

Duphamox© and Novamin were given intramuscular and the wound was covered with adhesive wound dressing. Fentanyl patch (50 µg) for postoperative pain management up to 72 h was applied to the shaved inner front limb.

The minipig was brought into an intensive care box while still being intubated and ventilated with O_2_-saturation continuously monitored. As soon as the animal was sufficiently and spontaneously breathing, the tube and all vascular accesses were removed. Finally, when the animal was awake, it was brought back to the pen and, if required, temporarily separated to avoid rank fights while still weakened.

To keep the physical burden to a minimum level the Control group did not undergo a sham procedure of pacemaker implantation, but animals were all together in the same facilities to reduce systemic bias.

### 2.2 Tachypacing protocol

The tachypacing protocol has been already described in our previous work (14). Briefly, after the pacemaker implantation, the animals had a recovery period of at least 7 days. Before starting the pacemaker, the pig was mildly sedated with a combination of Midazolam (0,35 mg/kg) and Ketamine (15 mg/kg) given intramuscularly. This dose led to a sedation that lasted approximately 45–60 min while providing oxygen (2–4 L/minute) and monitoring O_2_-saturation. This time-window was also used to perform echocardiography.

The pacemaker was programmed to asynchronously stimulate the right ventricle with two leads first at a rate of 180 bpm for 2-weeks, then at 200 bpm for 4-weeks. In detail, during the 2-weeks, each pacing lead was set to a frequency of 90 bpm with an interval of 333 ms between them to achieve an overall HR of 180 bpm. During the period of 4-weeks, each pacing lead was set to a frequency of 100 bpm for each lead with an interval of 300 ms to achieve an overall HR of 200 bpm. Correct stimulation frequency was verified by sonography and ECG. After examination, the animal was brought into the intensive care box until they could walk back into the stable without aid.

### 2.3 Echocardiography

An in-depth echocardiographic (Vivid I, GE Healthcare, Vienna, Austria) characterization of the model, conducted by experienced researchers and in compliance with the American Society of Echocardiography guidelines (15), has been already described in our previous work (14). The pacemaker was turned off 20 min prior examination to unmask myocardial stunning and give back autologous myocardial function at the present state. Three consecutive beats were measured and averaged. LV volumes and EF were assessed with the biplane method of disks in the apical 4- and 3-chamber view. LV dimensions were taken from end-diastolic and end-systolic images in parasternal long axis. The ventricular stiffness β was estimated by the method proposed by Kasner et al. (16) as $\beta =\;\frac{\log\;(DP\;÷30)}{\log\;(LVEDV\;÷V_{30})}$ where DP means diastolic pressure and V_30_ represents the end diastolic volume at which pressure equals 30 mmHg. Data was analyzed offline in a blinded fashion by 2 independent researchers.

### 2.4 Jacketed external telemetry and stress-level assessment

**Figure 1:**
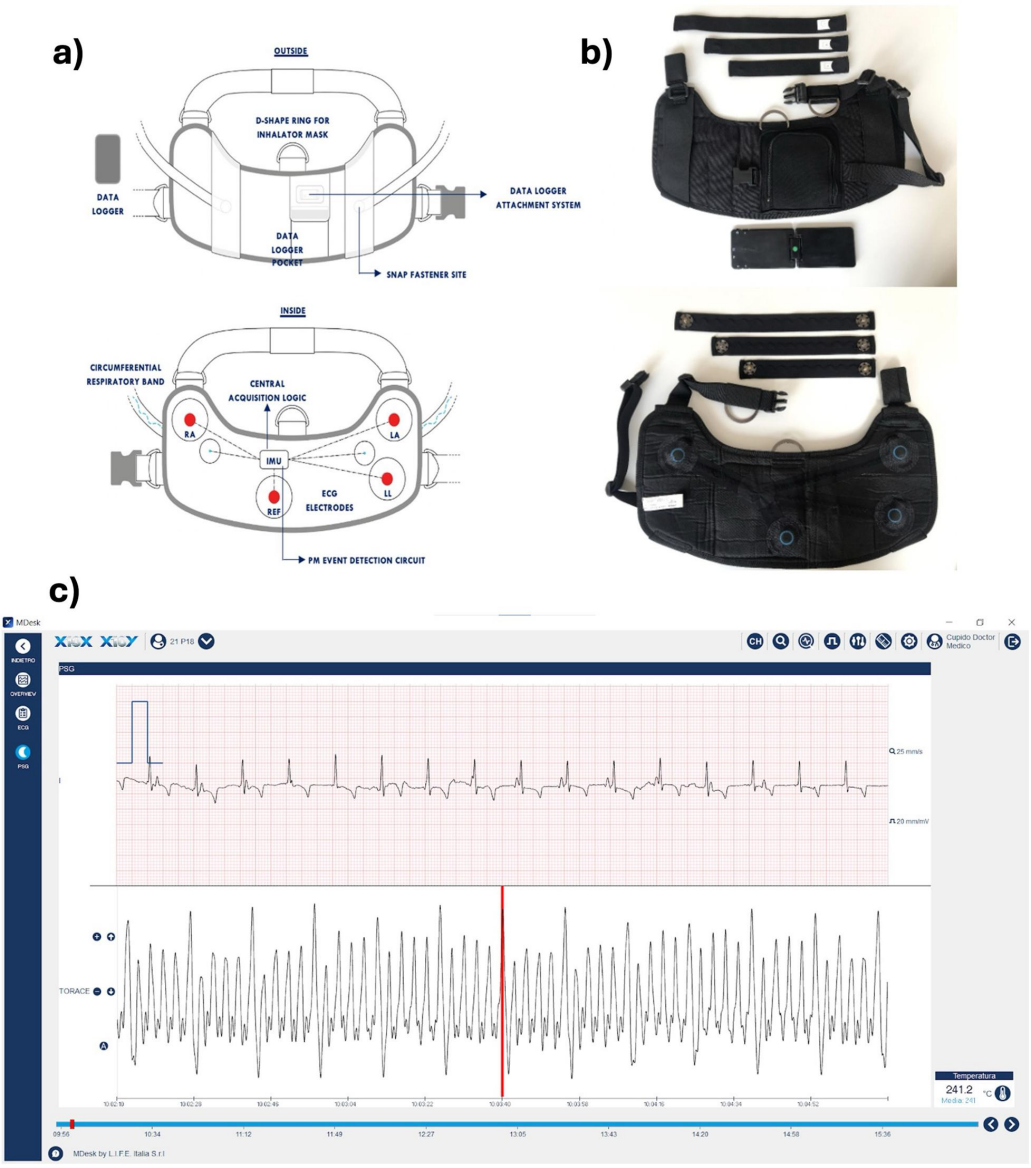
The developed model of jacketed external telemetry system (JET). **(a)** detailed drawing highlighting the sensors for data acquisition; **(b)** photograph of one prototype used in the trial. **(c)** ECG tracing and respiratory signal in the proprietary company software.

A jacketed external telemetry (JET) was developed and utilized with L.I.F.E. ITALIA S.R.L support (Figure 1). This non-invasive harness system continuously measured an ECG, the respiratory rate (RR), and animal movement to assess the daily activity of life (DAL). This wearable is available in different sizes and made of an ergonomic core with two adjustable bands to reduce the possibility of the device being removed from the animals. The bands were closed with a clip closure, Velcro-straps were avoided to prevent accumulation of dirt within the stables. A logger for data storing equipped with Bluetooth Low Energy and Wi-Fi connection was placed on the back of the jacket. This logger could store up to 64GB of data and record continuously for up to 12 h. Four carbon dry ECG electrodes gathered data at a frequency of 500 Hz, analogue to the three standard bipolar limb leads (I, II, III) as well as augmented leads (aVR, aVL, aVF). Before wearing the harness, the body surface in contact with the electrodes was shaved and a conductive gel applied to ensure sufficient signal quality. A circumferential extensometer was used to monitor extension of the rib cage during respiration with a frequency of 50 Hz. This band was available in three different sizes to assure optimal signal quality. The signal of the ECG and respiratory band could be monitored in real time via a dedicated app on smartphone (MyMedDy; L.I.F.E. ITALIA S.R.L.) and computer-based software (Mdesk; L.I.F.E. ITALIA S.R.L.). An inertial measurement unit measured DAL, acquiring signals from a 3-axis accelerometer, 3-axis gyroscope and 3-axis magnetometer. After data acquisition, the logger uploaded the data automatically via WIFI or via connection to a computer manually. The RR and DAL data was later analyzed offline. As the intervention group was paced at a fixed rate throughout the protocol, ECG data was not analyzed further.

Finally, to assess long-term cortisol and cortisone levels, hair samples were taken during the first four weeks of pacing and at sacrifice in 4 animals of the pacing group and in 1 control. The probes were analyzed by an external lab (DresdenLabservice GmbH) by method of liquid chromatography with coupled tandem mass spectrometry.

Additionally, the animals were monitored daily for signs of distress. This assessment was supported by a score-sheet containing pre-defined symptoms and cut-off values leading to closer monitoring or ultimately euthanasia under sedoanalgesia. The score-sheet can be seen in the supplemental material.

### 2.5 Invasive hemodynamics

An in-depth hemodynamic characterization of the model has been already described in our previous work (14). Briefly, animals were intubated after administration of 1 mg/kg propofol. Anesthesia was continued with 1%–1.5% isoflurane, 29 μg/kg/hour fentanyl and 0.2 µg/kg/hour pancouronium. Animals were ventilated with an FiO_2_ of 0.5, an I:E-ratio of 1:1.5, a positive end-expiratory pressure of 5 mmHg and a tidal volume of 10 mL/kg. If necessary, respiratory frequency was adjusted to maintain an end-tidal carbon dioxide partial pressure between 35 and 50 mmHg. A balanced crystalloid infusion (Sterofundin ISO, B. Braun Melsungen AG, Melsungen, Germany) was given at a fixed rate of 10 mL/kg/hour. The inner jugular vein and the common carotid artery were prepared on the opposite side of the pacemaker and 8F sheaths introduced via the Seldinger technique. Under fluoroscopic guidance a pulmonary artery flotation catheter (Swan Ganz, pediatric, 5F, Edwards Lifesciences CCO connected to Vigilance II; Edwards Lifesciences, Irvine, CA, USA) was placed in the pulmonary artery. A pressure-conductance catheter (5F, 12 electrodes, 7 mm spacing, MPVS Ultra; Millar Instruments, Houston, TX, USA) was inserted into the LV. The placement of the conductance catheter was checked under fluoroscope and online by inspecting the shape and direction of the pressure-volume loop (PVL) in real-time (Labchart 8, ADInstruments). After instrumentation, animals were allowed to stabilize for 30 min. Steady state hemodynamics were acquired in stable conditions over at least two respiratory cycles. After the experiment, the animals were sacrificed by infusion of a bolus of potassium chloride (100 mVal) and organs were explanted and weighed.

### 2.6 Data processing

Pressure data and time intervals were analyzed offline via CircLab software (custom made by Prof. P. Steendijk, Leiden University Medical Center, Leiden, The Netherlands) as described before (17). LV cardiac power output (LV CPO) was computed in Watt as mAP×(CO÷451).

Because JET was not available for every animal and at all times, we collected a fairly heterogenous dataset. As the RR data had a great number of artefacts, the data was split into segments of 10 s, median was computed, and datapoints above 40 were excluded. Then, data was summarized in hourly values. This generated dataset was visually inspected to identify time points capable to differ between day and night, as it was assumed that RR and DAL would decrease during nocturnal hours. Thus, day was defined as the time between 7 am and 6 pm and night between 8 pm and 7 am. The time interval from 6 pm to 8 pm was excluded to obtain a clearer separation between day and night.

Moreover, there was a change of the gyroscope sensor of the JET system during the trial, changing the scale of DAL data recorded (but not the overall biological effect) and potentially hindering the comparison between before and after modification. Therefore, to amend, each dataset was normalized to their corresponding control: a baseline value was computed as the mean of the control group for each dataset and every datapoint from the HF group was normalized to this value following the equation $\frac{\left(data\;point\;HF-baseline\;control\right)}{baseline\;control}$.

### 2.7 Statistical analysis

Data is presented as mean ± standard deviation if not stated otherwise. Normality was demonstrated by visual inspection of normal probability plots and Shapiro–Wilk test. Comparison between the two groups was computed with student´s *t*-test or Wilcoxon test. Comparison between two timepoints in one group was computed with student´s paired *t*-test or Wilcoxon test for paired tests. Telemetry data, cortisol and weight development data was analyzed using a linear and factorial mixed model to account for the heterogenous data structure, with the week as a fixed and the respected variable (RR, DAL) as a dependent variable. Extreme outliers were identified by z-score of 3.29 and excluded accordingly. Then, the two datasets were pooled and analysed in the aforementioned mixed model. A *p*-value < 0.05 was considered significant. For statistical calculations and graphical illustration, we used the software RStudio (Version 2022.12.0 + 353, PBC, Boston, MA).

## 3 Results

### 3.1 Protocol

The pacemaker was successfully implanted and operated in all animals without relevant complications and all subjects recovered within hours from the operation. Additionally, no wound infections were observed. The tachypacing protocol was well tolerated by 4 animals, without symptoms of disease or clinical signs of severe HF. In both Control and HF groups, sufficient image quality could be obtained in all animals. Table 1 reports the echocardiographic and biometric characteristics of the HF group after 6-weeks of tachypacing, showing the expected alterations due to the pathophysiology of HFrEF: highly depressed EF, increased end-systolic and -diastolic volumes.

**Table 1:** Heart failure group traits compared to control. Echocardiographic and weight characteristics of both Control and Heart failure groups on the day of sacrifice, after 6-weeks of tachypacing of the Heart Failure animals.

Characteristic	Control	HF
	(*n* = 4)	(*n* = 4)
Heart rate (bpm)	99 ± 21	82 ± 16
LV EF (%)	48 ± 2	21 ± 3***
LV end-systolic volume (mL)	16 ± 1	45 ± 8*
LV end-diastolic volume	35 ± 6	54 ± 6**
Stiffness coefficient beta	5.88 ± 0.03	5.95 ± 0.09
Bodyweight (kg)	29 ± 4	29 ± 2
Heart weight (g)	120 ± 15	157 ± 19*
Heart weight/bodyweight (g/kg)	4.41 ± 0.71	5.39 ± 0.79
Lung weight (g)	143 ± 14	209 ± 37*
Lung weight/bodyweight (g/kg)	5.25 ± 0.61	7.11 ± 0.86*

Values are mean ± standard deviation.

* *p* < 0.05.

** *p* < 0.01.

*** *p* < 0.001.

### 3.2 Development of compensated HFrEF

**Figure 2:**
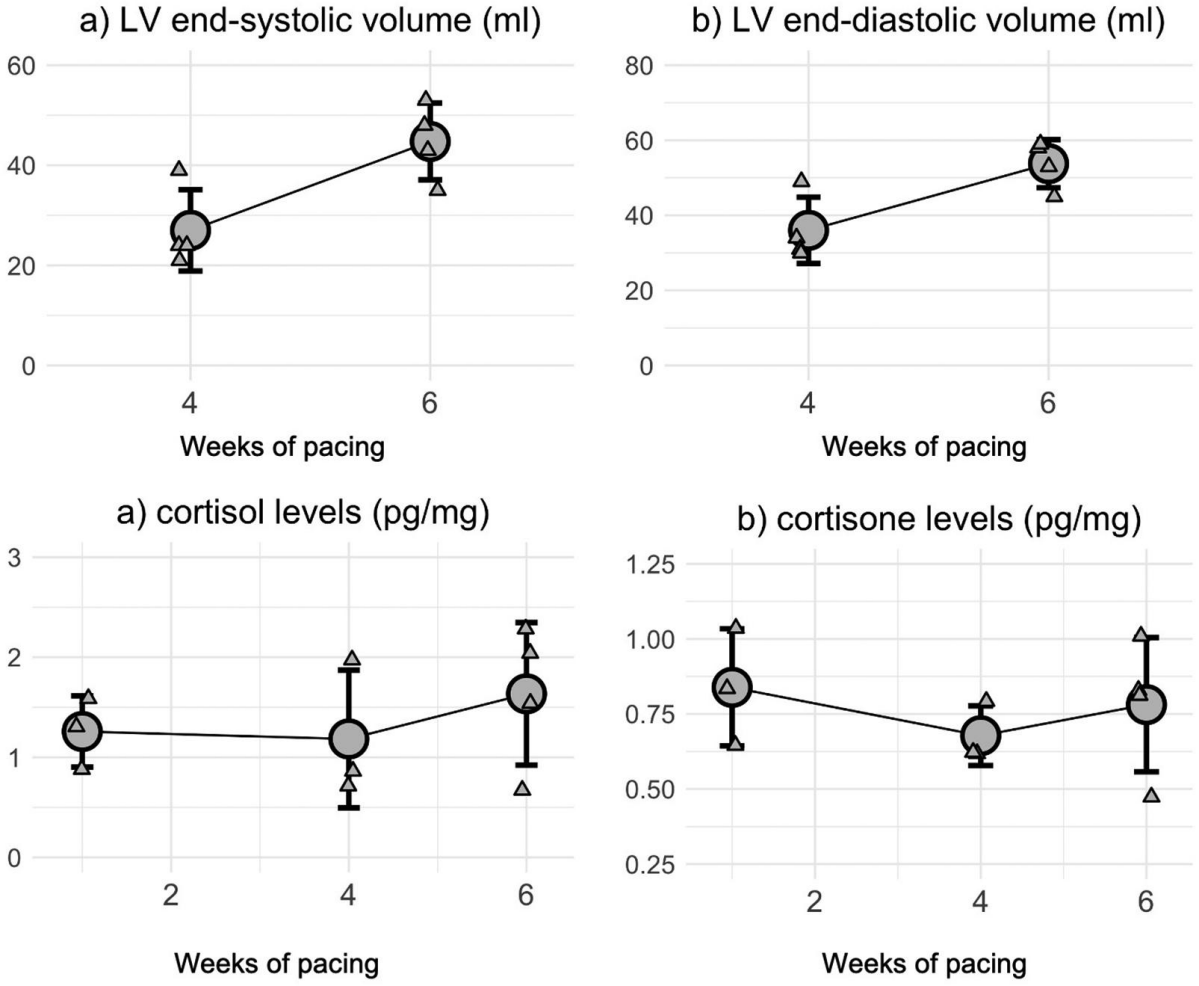
Heart failure progression and stress level during the last stage of the protocol. **(a)** LV end-systolic and **(b)** LV end-diastolic volumes show an increasing trend towards the end of the protocol, when the pig is stimulated at 200 bpm, hallmarks of chronic remodeling. **(c)** cortisol and **(d)** cortisone levels remain stable throughout the whole protocol.

As already described (13), pacing at 200 bpm for the last 4-weeks of the protocol led to a clear HFrEF phenotype. Particularly, from week 4-to-6, we denoted no alteration in heart rate (HR 82 ± 16 bpm, at both week 4 and 6) and LV EF (21 ± 2% vs. 21 ± 3.2%), although the latter was clearly depressed. However, we observed the worsening of the disease as both LV end-systolic (ESV) and -diastolic volumes (EDV) showed an increasing trend (ESV: 27 ± 8 mL vs. 45 ± 8 mL, *p* = 0.11; EDV: 36 ± 9 mL vs. 54 ± 6 mL, *p* = 0.25), indicating that the protocol affected the ventricular morphology (Figures 2a,b). Despite this, our protocol did not increase the animal stress levels, as both cortisol and corticosterone were stable throughout the study even when the pacing was increased to 200 bpm (Figures 2c,d). Representative original echocardiography movies can be seen in the online supplementary.

**Figure 3:**
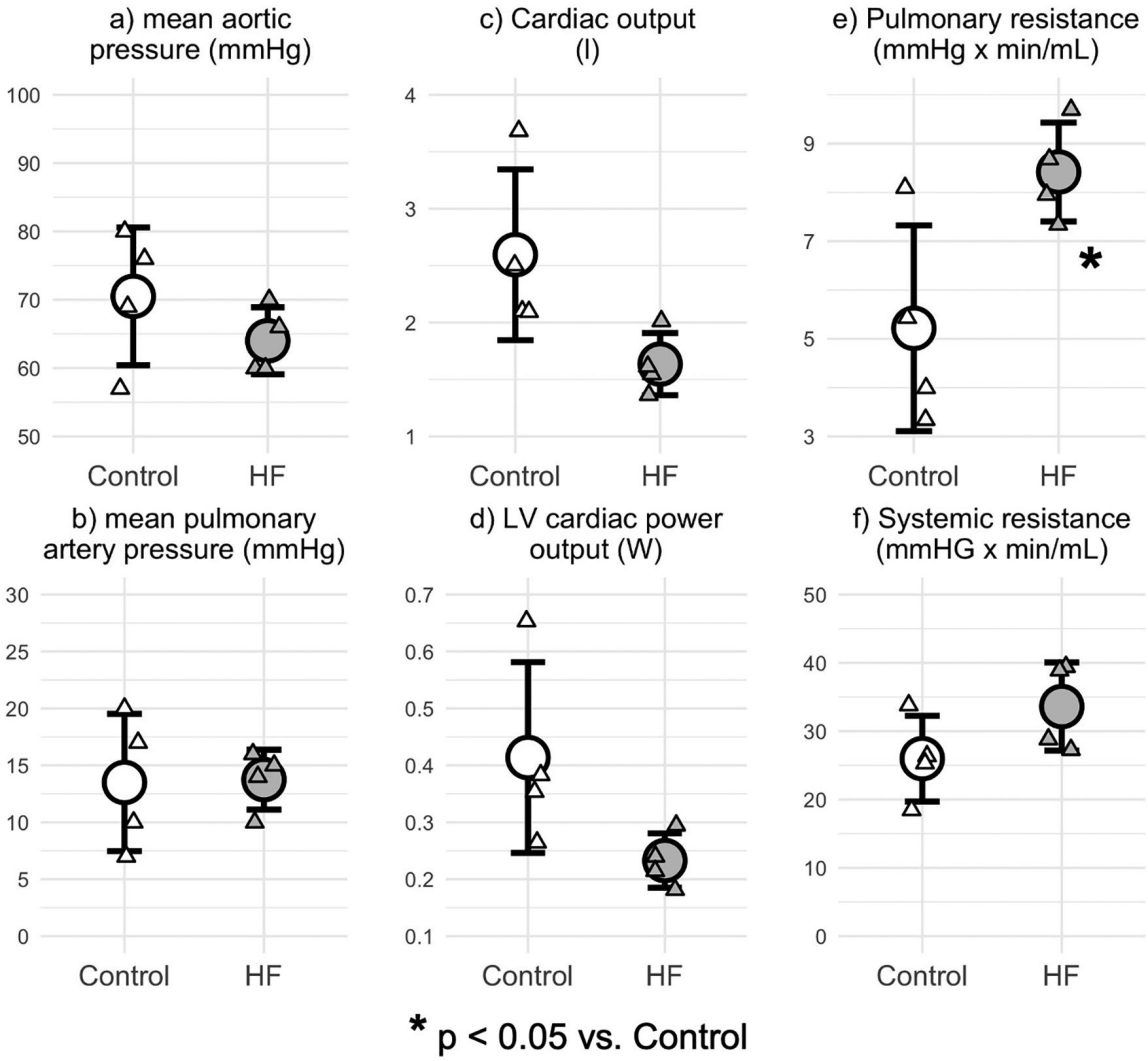
Invasive hemodynamic assessment proves the compensated HFrEF. **(a)** mean aortic pressure (mAOP) and **(b)** mean pulmonary arterial pressure (mPAP) were not altered due to tachypacing. **(c)** cardiac output and **(d)** left ventricle cardiac power output demonstrated the failure of the pump function, hallmarks of HFrEF. **(e)** pulmonary vascular resistance was significantly increased, in line with the expected pulmonary congestion in HFrEF. **(f)** systemic vascular resistance slightly increased but not significantly.

The invasive hemodynamic measurements at 6-weeks definitively demonstrated the establishment of compensated HF. In fact, the mean aortic pressure (mAOP) and mean pulmonary artery pressure (mPAP) was comparable between Control and HF groups (Figures 3a,b), despite both CO and LV CPO decreased in the failing group (Figures 3c,d). Systemic vascular resistance (SVR) was not significantly elevated in HF, whereas we observed a significant increase in pulmonary vascular resistance (PVR) due to presence of congestion in the failing group (Figures 3e,f).

### 3.3 Absence of stress or severe symptoms

**Figure 4:**
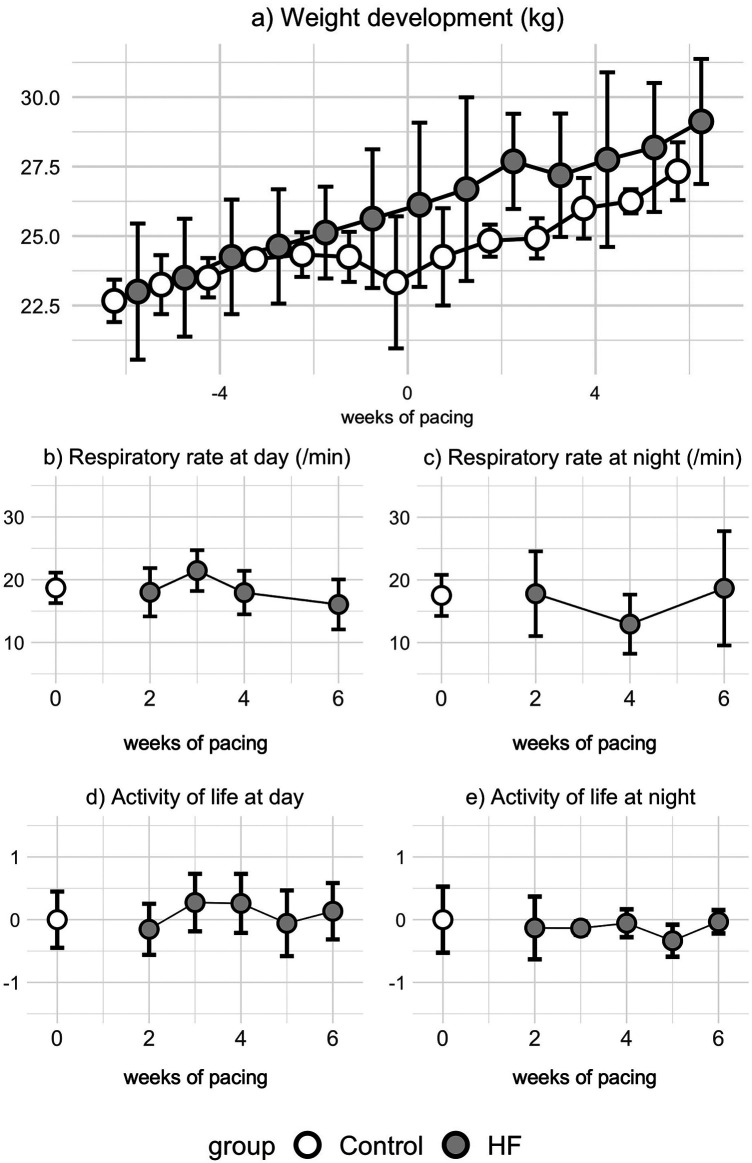
Assessment of distress. **(a)** weight-gain was not affected by the pacing protocol, as there was no difference between Control and HF. In the HF group, **(b)** respiratory rate at day or **(c)** at night did not show any alterations during the protocol. **(d)** Daily activity of life (e.g., moving around the stable, socializing with the other animals) during the day or **(e)** at night was stable throughout the pacing protocol.

As a sign of overall well-being, weight gain over the course of the protocol was comparable between Control and HF groups (Figure 4a). In detail, the HF group saw no decline in growth even after pacemaker implantation or in the final phase of tachypacing. The JET data further proved that the protocol was performed in the absence of stress. In fact, either during the day or at night, the RR was not altered with the development of HF (Figures 4b,c). Similar results were observed for the daily activity of life (e.g., moving around the stable, socializing with the other animals) during either day or at night (Figures 4d,e).

## 4 Discussion

In this study, we present a refined model of the classical tachypacing induced HF capable to induce DCM while reducing the stress for the animals.

### 4.1 Comparison to previous models

The main characteristics of the refinement of our protocol were the pacing frequency and its duration, with the pacemaker programmed to asynchronously stimulate the right ventricle with two leads at a rate of 180 bpm for 2-weeks and then at 200 bpm for 4-weeks. In fact, the classical tachypacing protocol would be to stimulate the heart at a 2–4-fold higher than physiological HR over a period of 3-to-5 weeks (8). This approach leads to a phase of compensated HF that rapidly decompensates towards the ending of the protocol. In several studies, animals develop severe symptoms of HF like dyspnea, cyanosis, peripheral and pulmonary edema, weight loss or lethargy (10, 12, 18–21). Powers et al, pacing at 210–240 bpm for ∼28 days suggest to monitor animals daily in the last week of decompensation and use these clinical signs to determine the efficacy of pacing to then initiate the performance of final experiments (10). This is further complicated as the onset of this rapid decompensation can vary from usually within a week to a range of 4–8 weeks (11, 22, 23). Additionally, the phase of rapid decompensation can lead to unexpected death of experimental animals, for example due to arrhythmia or severe heart failure (9, 24–26). Mortality can differ between 10% up to 37% opposed to the 0% mortality in this protocol. From the perspective of an experimental scientist this poses several difficulties as acute reaction to development of symptoms is necessary to perform experiments timely and complete datasets can be absent due to unplanned animal mortality.

The alterations we implemented in this study led to all the animals finishing the protocol after 6-weeks of pacing. The two-lead-pacemaker was successfully implanted in all animals and no postoperative infections were observed. As Möllmann *et al*. described, we opted to use the two-lead pacing because the resulting ventricular asynchrony leads to a higher impairment of cardiac function that lasts longer and is more reliable than conventional single-lead pacing (27). Additionally, commercially available single-lead pacemakers usually have a restricted maximal pacing rate lower than the required level to induce HF which can be circumvented with this dual-pacing method.

### 4.2 The refined model

Our revised pacing protocol clearly induced a stable, chronic, and compensated HFrEF. Echocardiography showed that LV EF was highly depressed in all HF animals, paired with chronic remodeling due to increased end-diastolic and -systolic volumes (i.e., LV dilatation), hallmarks of DCM. The invasive hemodynamic measurements proved the compensated nature of our model. In fact, in decompensated HF, mAOP crashes due to the overwhelming failure of the cardiac pumping ability that overcomes all the body's compensatory mechanism. Despite the impaired LV pump function in our model (i.e., CO and LV CPO), and evidence of pulmonary congestion as mirrored in increased lung weight [Table 1 and our previous work (14)], mAOP was maintained due to a compensatory increase of systemic vasoconstriction. Shortness of breath and fatigue are debilitating symptoms of decompensated HF because they directly result from the body's inability to meet its basic metabolic needs. Associated with decompensated HF is weight loss, another poor prognostic sign for patients and one of the most important humane endpoints in animal trials. Therefore, we monitor the weight-gain of the animals throughout the trial and developed a JET system to evaluate both breathing function and daily life activity in both Control and HF groups. The animals' weight-gain was comparable between the two groups. The JET system was well-tolerated by all animals, enabling the recording of more than 2.300 h of data. The analysis of respiratory rate, during either day or at night, indicated no systemic trend towards a higher level with HF progression. Same results were observed for the daily activity, showing no alterations at day or at night. Both cortisol and cortisone measurements from hair probes demonstrated the absence of severe symptoms and low level of stress, reinforcing our JET system findings.

In conclusion, we have successfully engineered a stable, chronic, and highly reproducible model of compensated HFrEF which is especially well-suited for chronic studies and hypothesis-testing of pharmacological or other interventions.

## 5 Limitations

This work is a sub-analysis of an extensive study conducted on this model. Indeed, a small sample size was included because the developed JET system was not employed for all the animals of the main study. This could be the reason for some parameters not reaching statistical significance. However, due to the known large effect size in clinically relevant parameters like LV ejection fraction, LV-dimensions, and contractility indices, we believe the number of animals to be adequate to demonstrate real effects. The large number of artifacts in the telemetry data limited its usability and the analysis of the DAL subset was further complicated by two different scales due to a mid-trial switch in the telemetry systems. However, standard data handling procedure were sufficient to obtain a coherent dataset. The tachypacing model has some known limitations itself as it cannot represent the various underlying causes for HF such as ischemia, valvular heart disease or elevated blood pressure. But it is well described in literature that the model of rapid pacing induced HF comes along with typical changes in neurohumoral changes (9, 28) underlining the clinical relevance and while we did not systematically examined activation of the sympathetic nerve or renin-angiotensin-aldosterone-system we showed in our previous work that our model leads to a significant rise of the N-terminal prohormone of brain natriuretic peptide (14). Further, it is well recognized that cardiac function typically recovers after 24–48 h after pacing induced HF which needs to be considered in the hemodynamic assessment. However, this should not account in our model as the pacing was stopped only for the short time required to perform echocardiography and invasive hemodynamics.

## 6 Conclusion

In this study we present a refined porcine model of tachypacing induced HF to reduce the stress on the animals caused by the acute decompensated phase. Our work shows a reduced disease burden compared to previous models, proved by unaffected cortisol levels, body weight, respiratory rate, and daily activity, despite the characteristic impaired pump function. For this reason we believe, our approach can be highly recommended as a preclinical platform for HFrEF and DCM in regard to further improve refinement in animal trials.

## Data Availability

The original contributions presented in the study are included in the article/Supplementary Material, further inquiries can be directed to the corresponding author.
