# Supplementary material for: A refined approach of the tachypacing porcine model of heart failure
Source: Front Cardiovasc Med. 2026 Mar 2;13:1726438. doi: 10.3389/fcvm.2026.1726438 (PMC12989331; doi:10.3389/fcvm.2026.1726438)
Supplement: Supplementary file 6 [file Datasheet2.pdf]

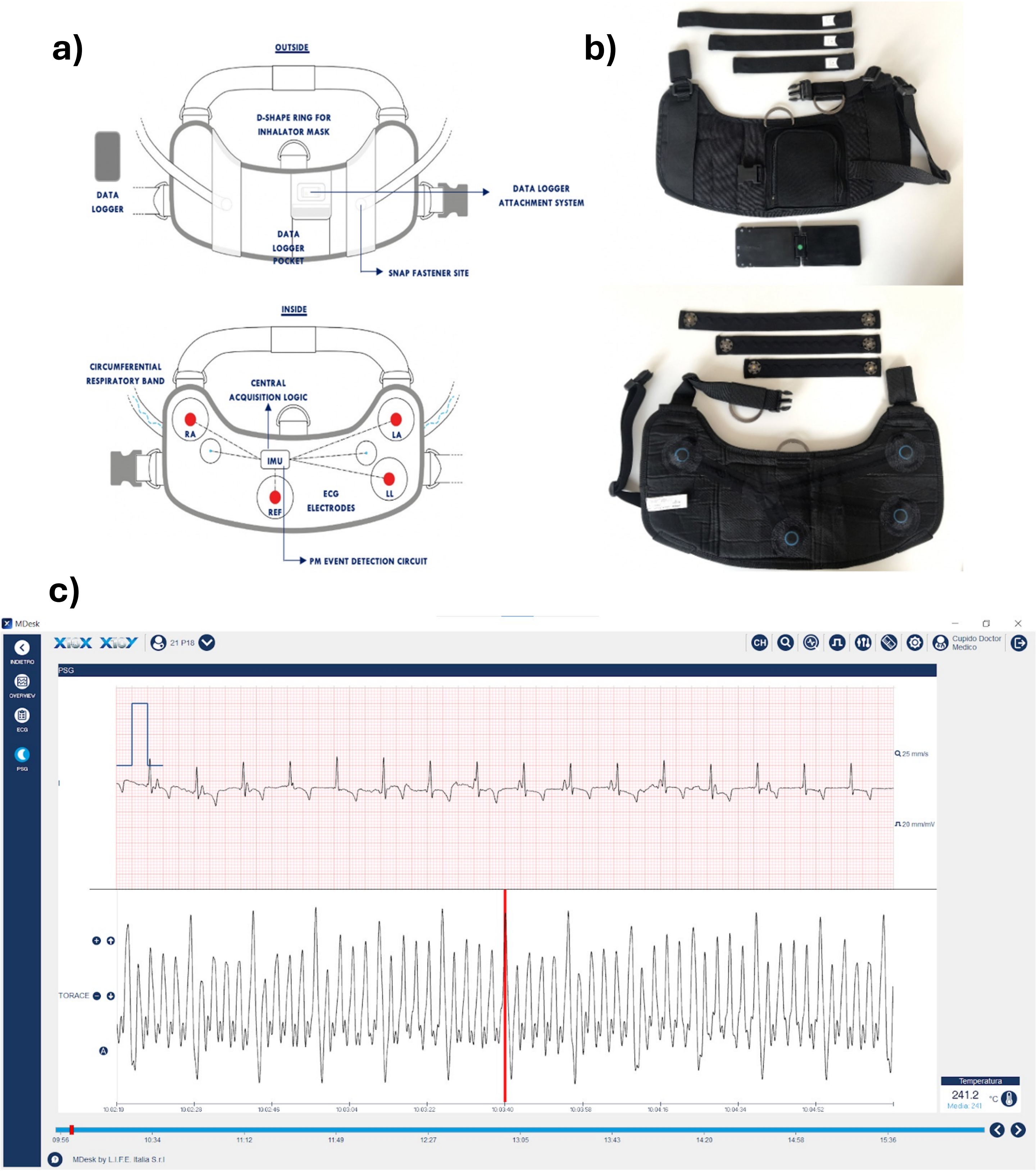
a)
OUTSIDE
D-SHAPE RING FOR INHALATOR MASK
DATA LOGGER
DATA LOGGER ATTACHMENT SYSTEM
DATA LOGGER POCKET
SNAP FASTENER SITE
INSIDE
CIRCUMFERENTIAL RESPIRATORY BAND
CENTRAL ACQUISITION LOGIC
RA
LA
IMU
LL
REF
ECG ELECTRODES
PM EVENT DETECTION CIRCUIT
b)
c)
MDesk
INDIETRO
OVERVIEW
ECG
PSG
21 P18
Cupido Doctor
Medico
PSG
25 mm/s
20 mm/mV
TORACE
09:56
10:34
11:12
11:49
12:27
13:05
13:43
14:20
14:58
15:36
Temperatura
241.2
°C
Media: 241
MDesk by L.I.F.E. Italia S.r.l

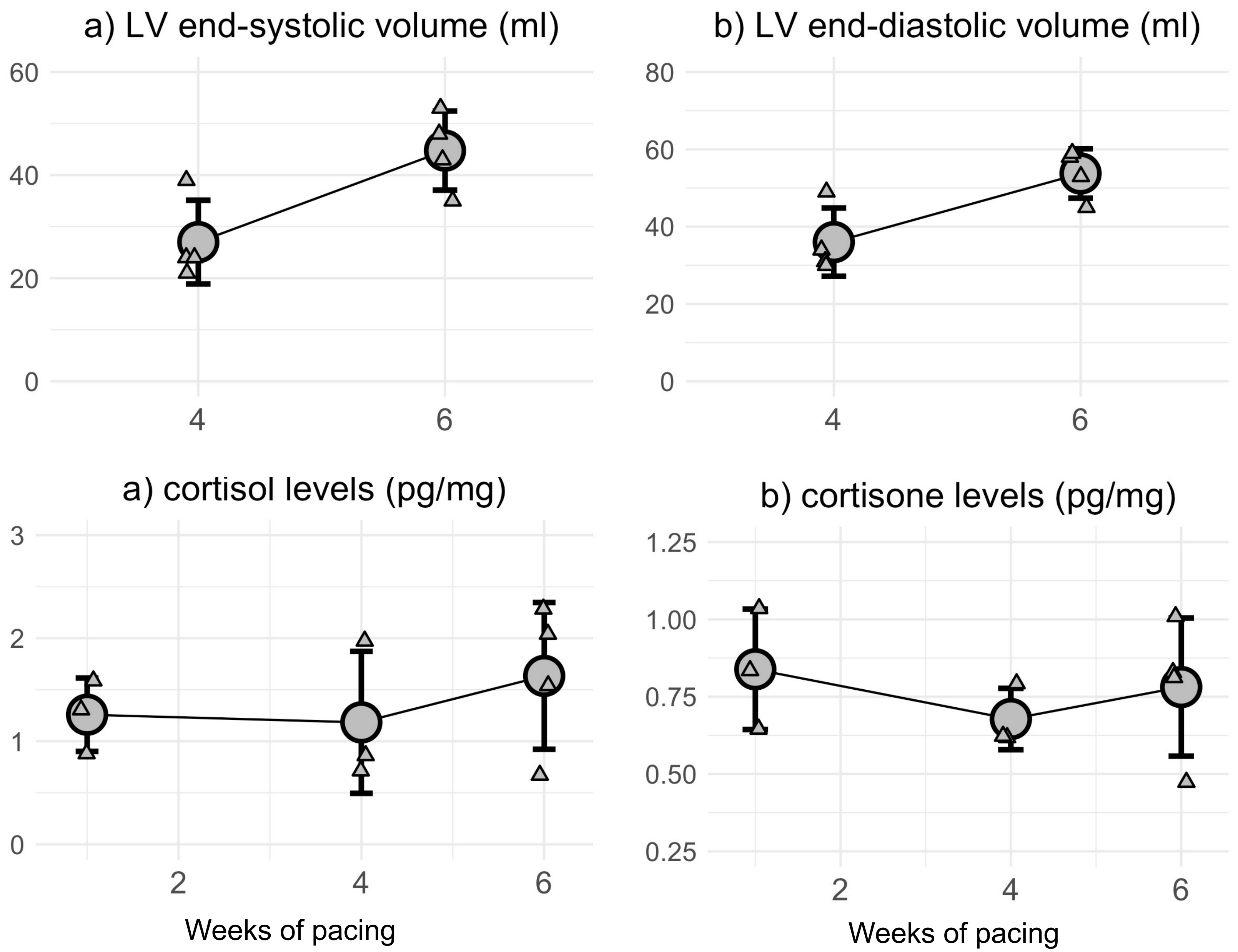
a) LV end-systolic volume (ml)
b) LV end-diastolic volume (ml)
a) cortisol levels (pg/mg)
b) cortisone levels (pg/mg)
60
40
20
0
80
60
40
20
0
4
6
4
6
3
2
1
0
1.25
1.00
0.75
0.50
0.25
2
4
6
2
4
6
Weeks of pacing
Weeks of pacing

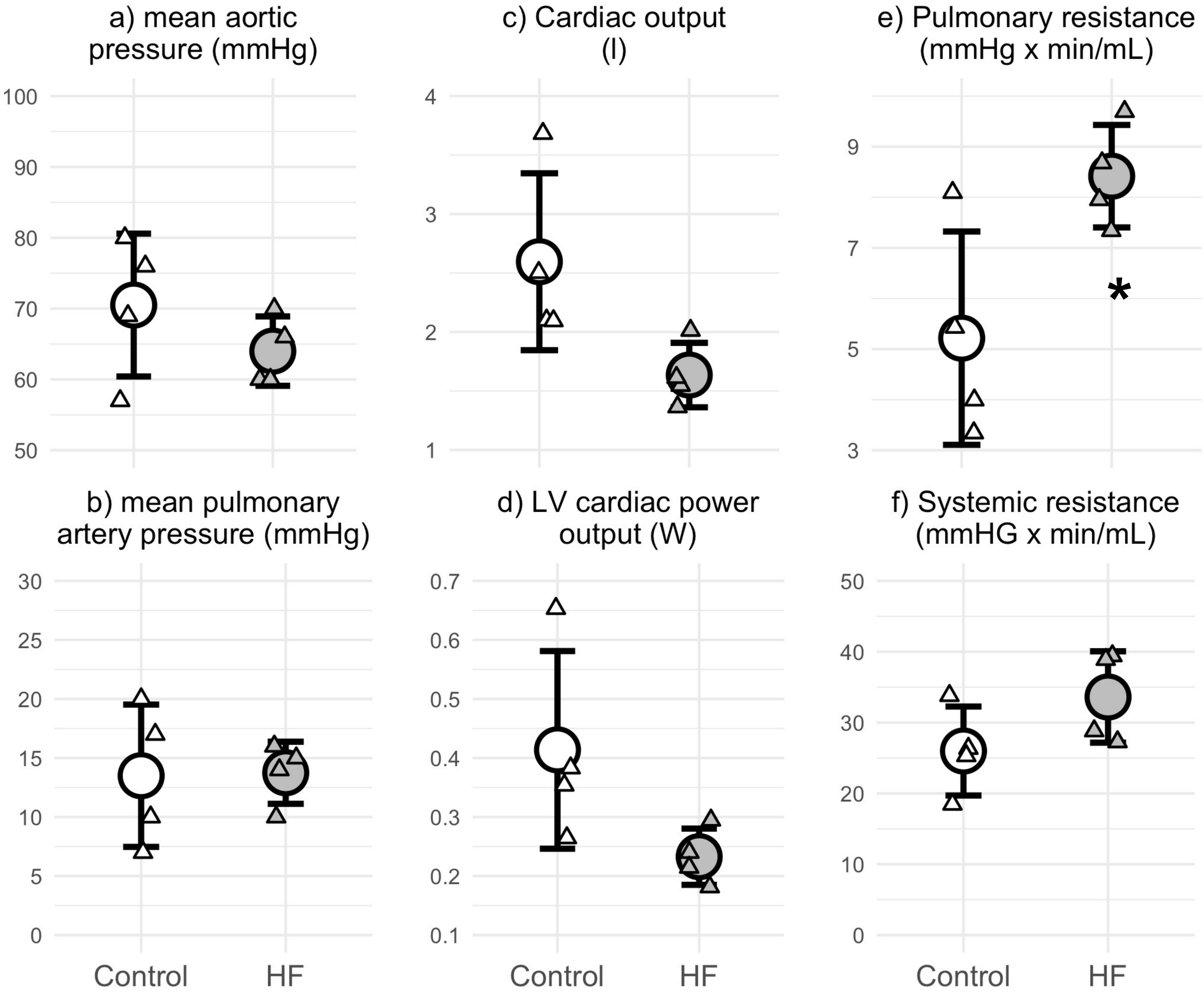

* $p < 0.05$ vs. Control

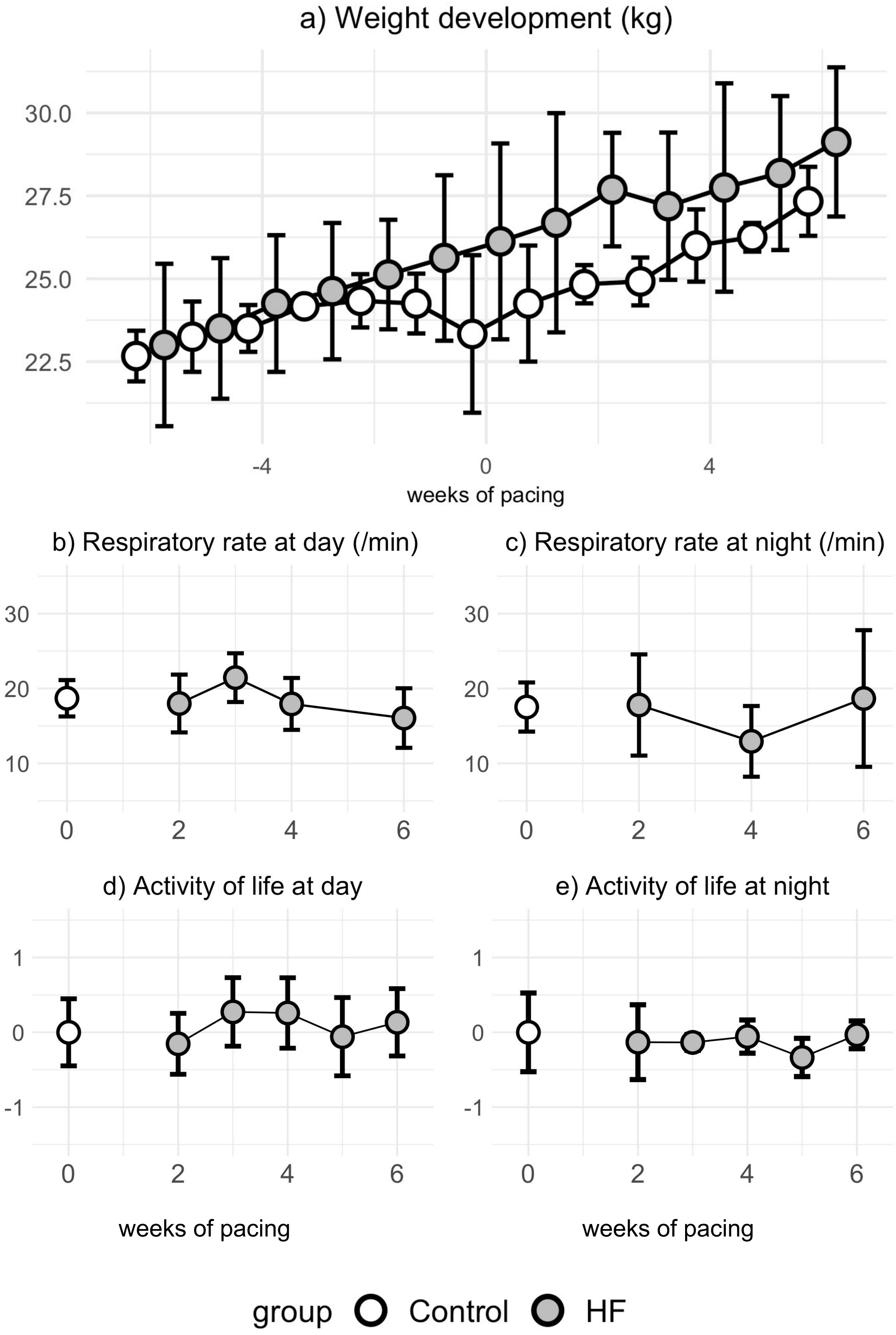
a) Weight development (kg)
30.0
27.5
25.0
22.5
-4
0
4
weeks of pacing
b) Respiratory rate at day (/min)
c) Respiratory rate at night (/min)
30
20
10
0
2
4
6
d) Activity of life at day
e) Activity of life at night
1
0
-1
0
2
4
6
weeks of pacing
weeks of pacing
group
Control
HF
